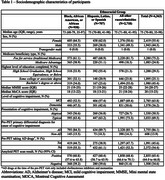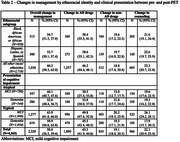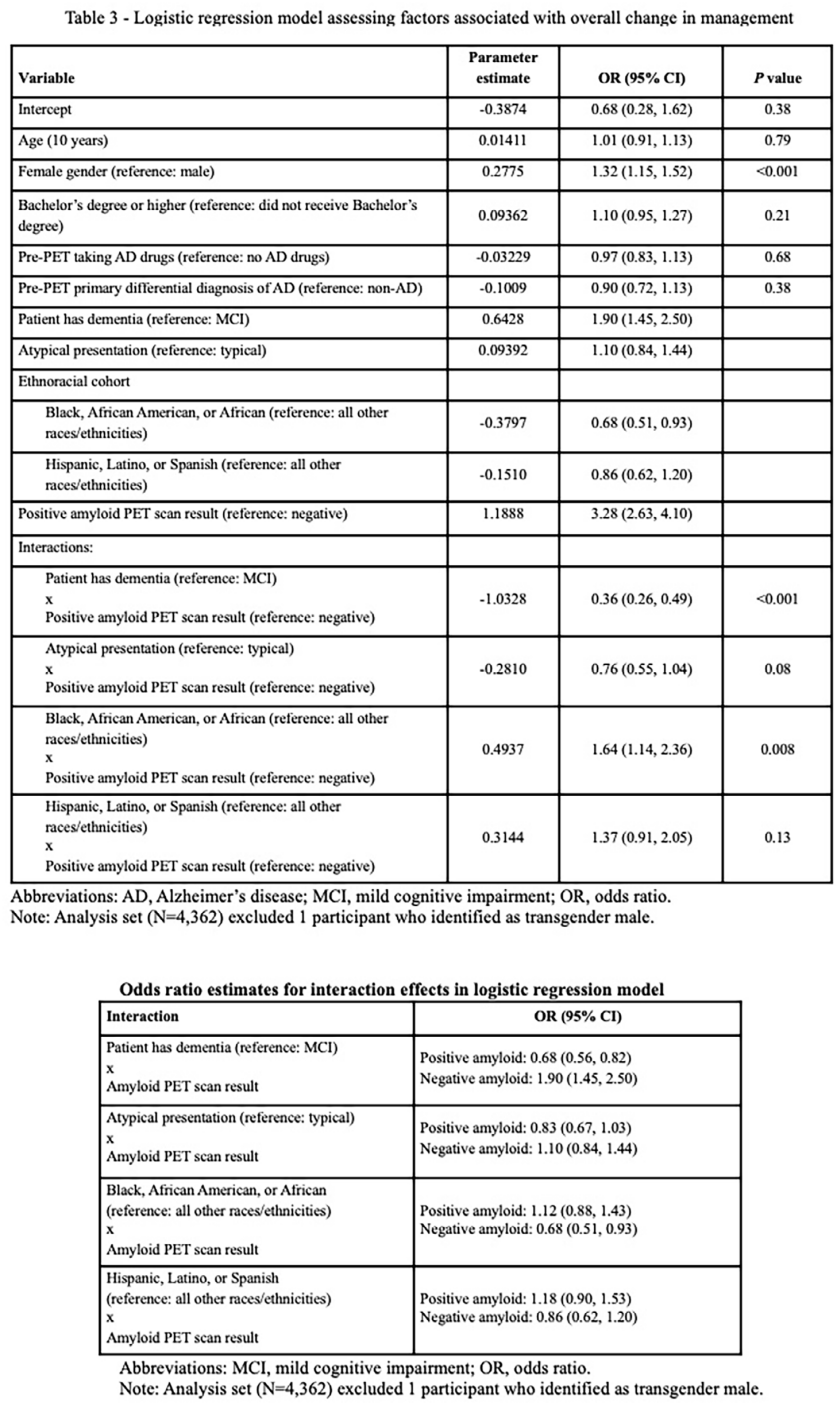# Amyloid PET and changes in clinical management among ethnoracially diverse and clinically atypical individuals: Findings from New IDEAS

**DOI:** 10.1002/alz70856_107734

**Published:** 2026-01-09

**Authors:** Charles C. Windon, Constantine Gatsonis, Justin Romanoff, Lucy Hanna, Peggye Dilworth‐Anderson, Maria C. Carrillo, Ilana F Gareen, Emily Glavin, Bruce E Hillner, Andrew March, Robert A. Rissman, Barry A. Siegel, Karen Smith, Rachel A. Whitmer, Christopher J. Weber, Consuelo H. Wilkins, Gil D. Rabinovici

**Affiliations:** ^1^ Memory and Aging Center, Weill Institute for Neurosciences, University of California San Francisco, San Francisco, CA, USA; ^2^ Dept. of Biostatistics, Brown University, Providence, RI, USA; ^3^ University of North Carolina, Chapel Hill, Chapel Hill, NC, USA; ^4^ Medical & Scientific Relations Division, Alzheimer's Association, Chicago, IL, USA; ^5^ American College of Radiology, Reston, VA, USA; ^6^ Virginia Commonwealth University, Richmond, VA, USA; ^7^ Alzheimer's Therapeutic Research Institute, University of Southern California, San Diego, CA, USA; ^8^ Mallinckrodt Institute of Radiology, Washington University School of Medicine, St. Louis, MO, USA; ^9^ Memory and Aging Center, UCSF Weill Institute for Neurosciences, University of California, San Francisco, San Francisco, CA, USA; ^10^ University of California, Davis, Davis, CA, USA; ^11^ Alzheimer's Association, Chicago, IL, USA; ^12^ Vanderbilt University Medical Center, Nashville, TN, USA

## Abstract

**Background:**

Amyloid PET use has been associated with change in clinical management among cognitively impaired older adults but various ethnoracial groups were underrepresented in prior studies. New IDEAS examined whether this association exists among cognitively impaired ethnoracially diverse Medicare beneficiaries and beneficiaries presenting with clinically “atypical” (non‐memory predominant) presentations of Alzheimer's disease (AD).

**Methods:**

New IDEAS was a national, multi‐site, prospective, longitudinal study that enrolled Medicare beneficiaries with mild cognitive impairment (MCI) or dementia who underwent amyloid PET scan as recommended by their treating dementia specialists at “real‐world” clinics. The study examined association between amyloid PET and subsequent change in clinical management within 90 days of PET.

Primary endpoint was change in management between pre‐ and post‐PET visits defined as a composite inclusive of change in AD and non‐AD drugs and change in counseling about safety and future planning. Proportions of change in management between pre‐ and post‐PET visit by group are reported as well as logistic regression examining association between composite change in management and multiple factors.

**Results:**

Median age of 4363 participants is 75 (range 35‐98) years and 55.4% are female, 63.8% having MCI and 65.4% (95% CI 64.0, 66.8) amyloid PET positive. The sample includes 938 (21.5%) Black/African American, 707 (16.2%) Hispanic/Latino, and 2718 (62.3%) other individuals with 1330 (30.5%) participants with atypical presentations of AD (Table 1).

Overall change in management occurred in 54.7% (95% CI 51.5, 57.9) of Black/African American, 53.7% (50.1, 57.4) of Hispanic/Latino, and 60.2% (58.3, 62.0) of other individuals. By clinical presentation, 44.1% (40.7, 47.6) of atypical clinical MCI, 52.6% (48.4, 56.7) of atypical dementia, 63.9% (61.8, 66.0) of typical MCI, and 59.9% (56.8, 62.8) of typical dementia participants had an overall change in management (Table 2). Logistic regression demonstrated significant interactions between Black/African American identity and positive amyloid PET scan (OR 1.64, 95% CI 1.14, 2.36; p 0.008) (Table 3).

**Conclusions:**

New IDEAS demonstrated that amyloid PET use among ethnoracially diverse Medicare beneficiaries and those with atypical and typical clinical presentations of AD leads to changes in clinical management, highlighting the value of this biomarker in a real‐world clinical care setting.